# Biophysical and Computational Studies of Membrane Penetration by the GRP1 Pleckstrin Homology Domain

**DOI:** 10.1016/j.str.2011.04.010

**Published:** 2011-09-07

**Authors:** Craig N. Lumb, Ju He, Yi Xue, Phillip J. Stansfeld, Robert V. Stahelin, Tatiana G. Kutateladze, Mark S.P. Sansom

**Affiliations:** 1 Department of Biochemistry, University of Oxford, Oxford OX1 3QU, UK; 2 Department of Pharmacology, University of Colorado Denver School of Medicine, Aurora, CO 80045, USA; 3 Department of Biochemistry and Molecular Biology, Indiana University School of Medicine–South Bend, South Bend, IN 46617, USA; 4 Department of Chemistry and Biochemistry and The Walther Center for Cancer Research, University of Notre Dame, Notre Dame, IN 46556, USA

## Abstract

The pleckstrin homology (PH) domain of the general receptor for phosphoinositides 1 (GRP1) exhibits specific, high-affinity, reversible binding to phosphatidylinositol (3,4,5)-trisphosphate (PI(3,4,5)P_3_) at the plasma membrane, but the nature and extent of the interaction between this bound complex and the surrounding membrane environment remains unclear. Combining equilibrium and nonequilibrium molecular dynamics (MD) simulations, NMR spectroscopy, and monolayer penetration experiments, we characterize the membrane-associated state of GRP1-PH. MD simulations show loops flanking the binding site supplement the interaction with PI(3,4,5)P_3_ through multiple contacts with the lipid bilayer. NMR data show large perturbations in chemical shift for these loop regions on binding to PI(3,4,5)P_3_-containing DPC micelles. Monolayer penetration experiments and further MD simulations demonstrate that mutating hydrophobic residues to polar residues in the flanking loops reduces membrane penetration. This supports a “dual-recognition” model of binding, with specific GRP1-PH-PI(3,4,5)P_3_ interactions supplemented by interactions of loop regions with the lipid bilayer.

## Graphical abstract

**Graphical Abstract:**

**Figure:**
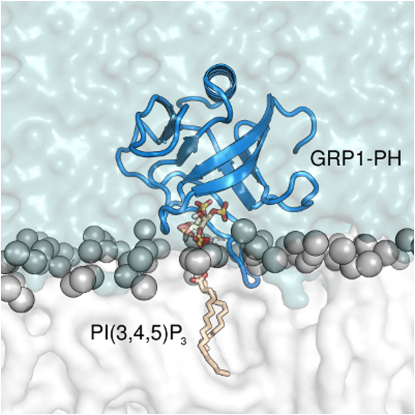

**Highlights:**

► Experiment and simulation to investigate the membrane-bound state of GRP1-PH ► GRP1-PH interacts with surrounding lipids in addition to PI(3,4,5)P3 ► Three loop regions near the binding site exhibit protein-lipid contacts ► This suggests a “dual recognition” model of PH binding to membranes

## Introduction

The successful recruitment of peripheral proteins to the cytoplasmic leaflet of the cell membrane in response to an external signal is a central step in many cell signaling pathways. Lipids have emerged as a key factor in the regulation of signaling (Cho and Stahelin, 2005; Wymann and Schneiter, 2008) and many signaling proteins possess a well-defined structural motif capable of binding to a specific lipid species, allowing the protein to preferentially target one particular component of the lipid bilayer. At least 11 of these lipid binding modules have now been identified (Lemmon, 2008; Stahelin, 2009) and among them the pleckstrin homology (PH) domain is one of the most common.

The PH domain is a highly structurally conserved domain of approximately 100 amino acid residues (Haslam et al., 1993; Mayer et al., 1993). This structural similarity is, however, somewhat remarkable given the low level of sequence identity among different PH domains (Lemmon et al., 1996). Many PH domains bind to phosphoinositides (PIs), a comparatively rare class of target lipid possessing a large negative charge, which arises from the presence of a variable number of phosphate group substituents on the inositol ring. Different patterns of phosphorylation at the 3′, 4′, and 5′ positions of the ring afford a total of seven distinct PI species. The low level of sequence homology between PH domains gives rise to a correspondingly broad spectrum of behavior, so while some PH domains are extremely specific and recognize only one of the seven species of PI, others are virtually unable to discriminate between them (Kavran et al., 1998; Manna et al., 2007).

The PH domain of the general receptor for phosphoinositides isoform 1 (GRP1-PH) is one of the most selective PH domains, reversibly binding to phosphatidylinositol (3,4,5)-trisphosphate (PI(3,4,5)P_3_) with pronounced specificity and high affinity (Klarlund et al., 1997). The concentration of PI(3,4,5)P_3_ in the cytoplasmic leaflet of the cell membrane is ordinarily very low, and so recruitment of GRP1 to the membrane surface is dependent upon the activity of class I phosphoinositide 3-kinases (PI3Ks), which are responsible for generating elevated levels of PI(3,4,5)P_3_ by phosphorylating the inositol head group of the more abundant phosphatidylinositol (4,5)-bisphosphate (PI(4,5)P_2_) (Cantley, 2002).

GRP1 is a member of the cytohesin family of proteins, which are small, phosphoinositide-dependent guanine nucleotide exchange factors (GEFs) that promote activation of Arf GTPases. Arf GTPases regulate several cellular processes including endocytosis and cytokinesis by switching between their active (GTP-bound) and inactive (GDP-bound) forms (D'Souza-Schorey and Chavrier, 2006), and they are thought to be particularly important in mediating the invasivity of tumor cells in breast and skin cancer (Tague et al., 2004). While the catalytic activity of GRP1 has been attributed to its Sec7 domain, recent experimental studies (DiNitto et al., 2007) have suggested that this activity is autoinhibited in the cytosol and that the protein only becomes catalytically competent when bound to the lipid bilayer. This underscores the importance of the GRP1 PH domain, since it is the high specificity and affinity of GRP1-PH for PI(3,4,5)P_3_ that is primarily responsible for driving the recruitment of its host protein to the surface of the plasma membrane. However, it is possible that when bound to the membrane, nonspecific interactions with the lipid molecules in close proximity may play a role in anchoring GRP1-PH to the lipid bilayer. It is the nature and strength of these additional protein-lipid interactions that are the subject of the present study.

**Figure 1:**
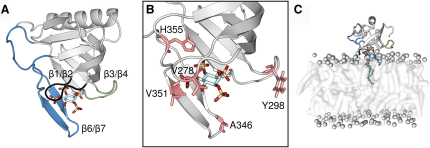
Structural Features of GRP1-PH (A) The three binding loops, β1/β2 (black), β3/β4 (green), and β6/β7 (blue), and their positions relative to the I(1,3,4,5)P_4_ head group. (B) The geometry of the I(1,3,4,5)P_4_ binding site, with the key amino acid residues referred to in the text shown as stick representations in pink. (C) The initial setup for the MD simulations, with the centers of the phosphate groups shown as gray spheres and the lipid bilayer depicted as a translucent white surface. Water molecules and ions are omitted for clarity. In all cases, the protein is shown as a ribbon diagram, and the I(1,3,4,5)P_4_ head group is shown as a stick model.

The canonical PH domain comprises a seven stranded β barrel capped at one end by an amphipathic α helix, while the other end is flanked by three loops (β1/β2, β3/β4, and β6/β7) that together form the PI binding site (Figure 1). The precise sequence of amino acid residues in these loops is a major factor in determining the specificity of the domain, and it has been observed that even small changes in this binding motif such as the addition or deletion of a single Gly residue can have a profound effect on the binding properties of the domain (Klarlund et al., 2000; Cronin et al., 2004). An unusual structural feature of GRP1-PH is the β6′/β6″ hairpin insertion, which lies within the extended β6/β7 loop. Comparative studies between different PH domains have suggested that the presence of this insertion in GRP1-PH is the factor that determines its pronounced PI(3,4,5)P_3_ specificity, as it “deepens” the ligand binding pocket and provides additional coordination for the 5′-phosphate (Ferguson et al., 2000). The position of the hairpin-insertion relative to the I(1,3,4,5)P_4_ head group in the crystal structure of GRP1-PH suggests that in the membrane-bound state this loop may also interact with the lipid bilayer. However, this remains only a conjecture as, although the binding of GRP1-PH to soluble inositol phosphates is now relatively well characterized, much less is known about the nature of the membrane-associated complex. In particular, the extent of the interaction between GRP1-PH and the lipid bilayer remains unclear. While surface plasmon resonance (SPR) experiments by Manna et al. (2007) and later by He et al. (2008) detected some membrane penetration, when Knight and Falke monitored the diffusion of individual GRP1-PH domains using single particle tracking in total internal reflection fluorescence microscopy (TIRFM) experiments, they found that, within error, the diffusion constant of the bound GRP1-PH-PI(3,4,5)P_3_ complex was the same as that of a free lipid (Knight and Falke, 2009). This was interpreted as indicating that there was comparatively little interaction between the bound PH domain and the adjacent membrane, since such an interaction would be expected to exert a viscous drag force on the bound complex and slow its diffusion rate.

Molecular dynamics (MD) simulations are a powerful computational tool for investigating protein-lipid interactions (Lindahl and Sansom, 2008). Previous MD simulation studies reflect the diversity of membrane-binding domains, and include MD simulations of membrane-binding modules such as N-BAR (Blood and Voth, 2006; Arkhipov et al., 2008), C2 (Jaud et al., 2007; Manna et al., 2008; Lai et al., 2010), GLA (Ohkubo and Tajkhorshid, 2008), PH (Psachoulia and Sansom, 2008), talin (Kalli et al., 2010), and PX and FYVE (Psachoulia and Sansom, 2009). In the current study, we examine the interaction between GRP1-PH and the membrane, focusing on the relative importance of the specific, PI-mediated interactions and the nonspecific interactions with the lipid molecules of the adjacent bilayer. Our approach combines MD simulations with experimental biophysical techniques including NMR spectroscopy and monolayer penetration studies. The outcome is a more comprehensive description of the nature of the membrane-associated state of GRP1-PH complexed with PI(3,4,5)P_3_. In particular, our results support a “dual-recognition” model of GRP1-PH binding to membranes, combining specific electrostatic interactions between basic side chains and the PI(3,4,5)P_3_ head group with penetration of the bilayer core by a hydrophobic loop. This is consistent with the membrane insertion behaviors displayed by other lipid-binding modules such as Phox-homology (PX) domains (Lemmon, 2008). Membrane insertion of the PH domain of phospholipase C-δ_1_ has also been observed (Flesch et al., 2005) though the authors concluded that this may be because this domain exhibits different membrane binding modes depending upon the composition of the lipid bilayer.

## Results

### MD Simulations of the Membrane-Bound GRP1-PH-PI(3,4,5)P_3_

To generate a suitable initial configuration of the GRP1-PH-PI(3,4,5)P_3_ complex at the membrane surface, it was necessary to place the crystal structure of the protein incorporating the I(1,3,4,5)P_4_ head group at an appropriate position and orientation relative to the lipid bilayer. The orientation of PI head groups at the surface of lipid bilayers has recently been studied extensively by Pastor and co-workers (Li et al., 2009) using MD simulations and finite-difference Poisson Boltzmann calculations. Li et al. found that the I(1,3,4,5)P_4_ head group sampled a range of tilt-twist (θ-φ) angles at the membrane surface, but that some orientations were preferred. We used these as a guide when positioning the ligand-bound GRP1 PH domain relative to the surface of the membrane, matching the orientation of its bound I(1,3,4,5)P_4_ head group to the preferred θ-φ angles determined by Li et al. (see Experimental Procedures for more details).

**Figure 2:**
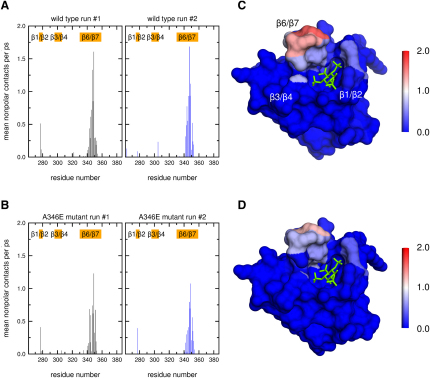
Membrane Penetration of GRP1-PH Observed during the MD Simulations (A) Mean number of nonpolar protein-lipid contacts per residue per ps over the 100 ns for the two wild-type protein simulation (one simulation in black and the second simulation in blue). (B) The same measure for the two A346E mutant simulations. (C) and (D) show these contacts projected on to the molecular surface of the protein for the wild-type and A346E mutants respectively, illustrating the decrease in penetration depth of the β6/β7 loop upon mutation. The color scale corresponds to the plots in (A) and (B) and shows the mean number of nonpolar protein-lipid contacts per residue per ps, varying from 0 (blue) to 2 (red). Nonpolar contacts are defined as the number of POPC tail carbon atoms within 4 Å of a protein heavy atom. See also Figure S3.

To explore the extent of the interaction between GRP1-PH and the lipid bilayer, we first performed two 100 ns equilibrium MD simulations of the membrane-bound GRP1-PH-PI(3,4,5)P_3_ complex using two different sets of initial velocities. These simulations revealed an array of contacts between the protein and the lipids of the adjacent bilayer, showing that the β1/β2, β3/β4, and β6/β7 loops flanking the binding site are well positioned to interact with the membrane. To assess the degree of membrane penetration by each of the three loops, we analyzed the nonpolar protein-lipid interactions (Figure 2), which show that the residues within the β1/β2 and β3/β4 loops form transient nonpolar contacts with the POPC carbon tails over the course of the trajectory. The β6/β7 loop on the other hand experiences a much more sustained interaction with the lipid acyl chains throughout the simulation, indicative of a greater degree of penetration into the hydrophobic core of the membrane. Thus, the extension to the core β barrel provided by the hairpin insertion in the GRP1 PH domain not only deepens the PI(3,4,5)P_3_ binding pocket but also appears to provide a framework for more extensive interactions with the surrounding membrane lipids. Several residues in GRP1-PH interact directly with the I(1,3,4,5)P_4_ head group in the crystal structure (Lietzke et al., 2000), and this extensive network of interactions is preserved throughout both simulations of membrane-bound GRP1-PH. Analysis of the hydrogen-bonding interactions between the amino acid residues lining the binding pocket of GRP1-PH and the phosphate groups of PI(3,4,5)P_3_ detected hydrogen bonds between PI(3,4,5)P_3_ and the following amino acid residues: K273; G276; R277; V278; T280; K282; R284; Y295; R305; K343; and H355. This is in excellent agreement with the hydrogen-bonding pattern found in the crystal structure, with only the hydrogen bond donated by N354 to the 5′-phosphate not detected. This indicates that the binding mode adopted by the soluble I(1,3,4,5)P_4_ head group is preserved in the membrane-associated state when GRP1-PH is bound to PI(3,4,5)P_3_.

### NMR Spectroscopy of PI(3,4,5)P_3_-Bound Protein in DPC Micelles

**Figure 3:**
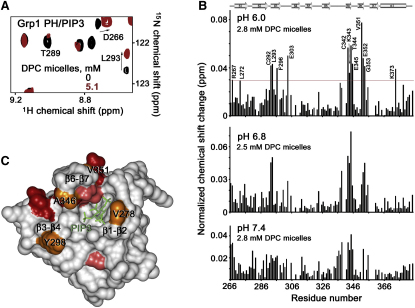
Association of the PI(3,4,5)P_3_-Bound GRP1-PH with Membrane-Mimicking DPC Micelles Monitored by NMR (A) Superimposed ^1^H, ^15^N HSQC spectra of the PI(3,4,5)P_3_ (0.4 mM)-bound PH domain (0.2 mM) in the DPC-free state (black) and in the presence of 5.1 mM DPC micelles (red) collected at pH 6.8. (B) The histograms show normalized (Grzesiek et al., 1996) chemical shift changes induced in the backbone amides of the PI(3,4,5)P_3_-bound GRP1 PH domain by DPC micelles at indicated pH. Significant changes in resonances are judged to be greater than the average plus one standard deviation (red line). (C) Residues that display significant changes in chemical shift are colored in red and pink for large and medium changes, respectively. Mutated residues are orange. The head group of PI(3,4,5)P_3_ is shown as a stick model and colored green.

To test the prediction that it is these regions of GRP1-PH that interact with the lipid bilayer, we investigated the association of the PI(3,4,5)P_3_-bound protein with membrane-mimetic DPC micelles using NMR spectroscopy. The ^1^H, ^15^N HSQC spectra of the ^15^N-labeled PH domain in complex with PI(3,4,5)P_3_ at a 1:2 protein-to-lipid ratio were collected as DPC micelles were titrated in (Figure 3). Substantial changes in amide resonances of R267, L272, C292, L293, F296, E303, C342, K343, T344, E345, V351, E352, G353, and K373 were observed, indicating that these residues are directly or indirectly involved in the interaction with the micelles. The majority of the most perturbed residues are located in the β1 and β3 strands and in the β3/β4 and β6/β7 loops of the PH domain. The magnitude of the perturbation in chemical shift is particularly large in the region corresponding to the β6/β7 loop, suggesting that of the three loops the β6/β7 loop interacts with the lipid environment to the greatest extent, in good agreement with the MD simulations. The NMR studies also indicate that monolayer penetration of GRP1-PH is pH dependent, as the magnitude of the NMR perturbations in the PI(3,4,5)P_3_-bound GRP1 PH domain induced by DPC micelles is increased by lowering the pH. This builds on previous work (He et al., 2008), which established that protonation of the H355 residue increases the binding affinity of the protein.

### Steered Molecular Dynamics Simulations of Dissociation of GRP1-PH from a Membrane

**Figure 4:**
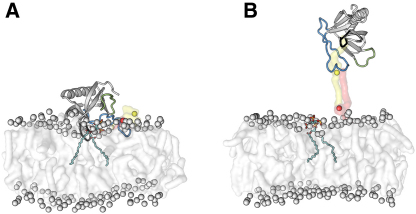
Simulation Snapshots of GRP1-PH Being Pulled from the Bilayer Surface (A) Snapshot at *t* = 0 ns for the neutral H355 case. (B) Snapshot at *t* = 36 ns, again for the neutral H355 case. The interaction between the β6/β7 loop and the membrane lipids is sufficiently strong to completely remove a lipid from the bilayer (shown in yellow) at this pulling velocity, while another lipid (shown in red) is partially extracted. See also Figure S4.

On the basis of the NMR results, we wished to investigate the relative strength and pH dependence of the interactions between each of the three binding loops and the PI(3,4,5)P_3_-containing lipid bilayer. To this end, we used steered molecular dynamics (SMD) simulations to probe the likely mechanism of dissociation of the complex. Thus, the bound PH domain was “pulled” away from the ligand and the surrounding lipid bilayer by a force applied parallel to the z axis, i.e., perpendicular to the plane of the membrane (Figure 4). The sequence in which the protein-ligand and protein-lipid contacts are broken as the protein is pulled away allows us to explore the relative contribution of each of the loop regions in sustaining the complex.

By performing two SMD simulations, one with the side chain of H355 in a neutral state and another with the side chain in a protonated state, we can mimic the effect of a change in pH and assess how this affects the interaction between the protein and the ligand or the membrane. Each pulling simulation was performed twice, once at a pulling speed of 1.0 Å/ns over a period of 40 ns and once at a pulling speed of 0.5 Å/ns over a period of 80 ns, in order to assess the reproducibility of the results. This gave a total of four SMD simulations performed under different conditions.

The β6/β7 loop that penetrated the lipid bilayer to the greatest extent in the equilibrium MD simulations is sufficiently tightly bound to the lipid bilayer that it is able to extract POPC lipids upon dissociation of the complex in the SMD simulations (Figure 4). This effect is even more pronounced when H355 is protonated.

**Figure 5:**
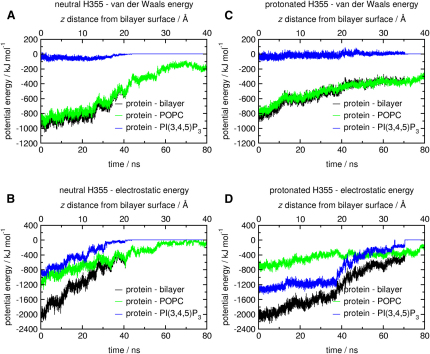
Potential Energy of the Protein-Membrane Complex over the SMD Simulations Short-range components of the van der Waals and electrostatic contributions to the potential energy are shown for the slower SMD simulations, of duration 80 ns and a pulling rate of 0.5 Å/ns. (A) van der Waals potential energy and (B) electrostatic potential energy for the SMD simulation with H355 in a neutral state. (C) van der Waals potential energy and (D) electrostatic potential energy for the SMD simulation with H355 in a protonated state. See also Figures S1 and S2.

The electrostatic and van der Waals contributions to the potential energy of the complex over the course of the SMD simulations were extracted and are shown in Figure 5 for the slower pulling simulations, with a pulling rate of 0.5 Å/ns for 80 ns (see Figure S1 available online for the faster pulling simulations, with a pulling rate of 1.0 Å/ns for 40 ns). The lipid bilayer provides the dominant contribution to the van der Waals component of the potential energy in both cases. The van der Waals component remains nonzero at the end of both the simulations despite the protein having been pulled away from the membrane due to the extraction of lipids from the bilayer as discussed above, which remain bound to the protein as it is pulled away (Figure 4). Interestingly, in the case where H355 was in a neutral state, the protein-PI(3,4,5)P_3_ interaction and the protein-POPC interaction both contribute in approximately equal measure to the overall electrostatic interaction between the protein and the membrane. This is likely due to multiple interactions between the protein and the phosphatidylcholine head groups in the lipid bilayer. However, when H355 is protonated, the protein-PI(3,4,5)P_3_ interaction dominates. As before, the component of the electrostatic potential energy does not fall to zero over the course of the simulation, again as a result of the extraction of lipid molecules from the bilayer. Pulling the protein over the same distance at a faster rate (Figure S1) gives similar results.

The considerable contribution to the van der Waals and electrostatic potential energy of the protein-membrane complex provided by the POPC lipids suggests that the lipid bilayer plays an important supporting role in the membrane interaction of GRP1-PH. Since the H355 residue lies within the β6/β7 loop, which experienced the most sustained interaction with the lipid bilayer during the MD and SMD simulations, we speculate that protonation of this residue plays a dual role in increasing the binding affinity of the complex by not only reinforcing the specific interaction between H355 and PI(3,4,5)P_3_, but also by extending the residence time of the β6/β7 loop in the lipid bilayer through more extensive electrostatic interactions with the head groups of the surrounding lipid bilayer. In this respect, the protonated H355 residue could be said to act as a “latch” mechanism, which prolongs the interaction between the loop and the membrane as the protein is pulled away.

Similar SMD simulations were also performed for the isolated protein with the I(1,3,4,5)P_4_ head group in solution, with no bilayer present. Again, simulations were carried out for the two cases of neutral and protonated H355. Comparison with the previous simulations in which the membrane was present shows that the inclusion of the lipid bilayer appears to prolong the contacts between the PI(3,4,5)P_3_ head group and the protein (Figure S2), again pointing toward a potential “dual-recognition” mechanism where the lipid bilayer complements the specific interaction between GRP1-PH and PI(3,4,5)P_3_.

### Monolayer Penetration of GRP1-PH

**Figure 6:**
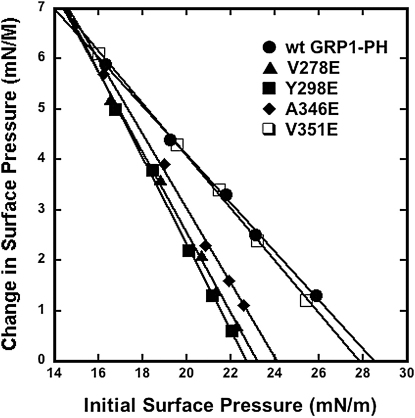
Monolayer Penetration Experiments of GRP1-PH and Respective Mutations Insertion of the wild-type GRP1 PH domain (filled circles), V278E (filled triangles), Y298E (filled squares), A346E (filled diamonds), and V351E (open squares) mutations into a POPC/POPE/PI(3,4,5)P_3_ (77:20:3) monolayer in a subphase of 10 mM HEPES/0.16 M KCl (pH 7.4) monitored as a function of π.

To further define the role of the three regions in interacting with and inserting into the lipid bilayer, the hydrophobic residues of each loop (V278 of β1/β2, Y298 of β3/β4, A346, and V351 of β6/β7) were each replaced with a Glu residue and the corresponding mutant proteins were evaluated by monolayer penetration experiments (Figure 6). The wild-type PH domain penetrated up to 28 mN/m at pH 7.4 as previously reported (He et al., 2008), and the V351E mutation displayed a similar degree of penetration. However, the V278E, Y298E, and A346E mutants all had exhibited reduced penetration, with π_c_ values between 23 and 24 mN/m for all three mutations.

As the β6/β7 loop penetrated most deeply into the bilayer in the MD simulations of the wild-type protein, we performed simulations of the A346E mutant to test how this might alter the degree of membrane insertion. Again, we performed a 100 ns simulation of the mutant complex and also repeated this simulation using different initial velocities. The array of contacts between GRP1-PH and PI(3,4,5)P_3_ observed for the wild-type simulation is conserved in the mutant. However, the nonpolar contacts between the β6/β7 loop and the lipid tails in the mutant become less frequent in comparison to the wild-type simulations (Figure 2).

## Discussion

Results from our MD simulations indicate that the GRP1 PH domain is able to penetrate the membrane, with the three loops flanking the binding site together forming a dynamic yet sustained array of contacts with the membrane lipids. However, we note that MD simulations do suffer from some limitations, which we discuss below.

Our docked structure is based on the GRP1-PH crystal structure superimposed onto a POPC lipid bilayer, with the head group orientation matched to that observed in previous MD simulations of PI lipids (see Experimental Procedures). This superimposition of the ligand-bound crystal structure onto a lipid bilayer also determined the initial orientation of the protein relative to the bilayer surface. However, the crystal structure of the protein bound to a soluble inositol phosphate may not be representative of the membrane-bound conformation. The protein has to first approach the membrane and then bind to the surface in vivo, a process thought to be dominated by electrostatic interactions, and this may involve conformational changes not captured by our static superimposition of the crystal structure onto the PI(3,4,5)P_3_-containing membrane. This may be particularly true of the three unstructured flanking loops. Nonetheless, we are encouraged by the good agreement with the experimental data which suggests we have determined the correct overall orientation of the protein relative to the bilayer.

To test whether or not our positioning of the protein relative to the surface of the lipid bilayer was appropriate, we estimated single-molecule lateral diffusion constants for GRP1-PH from the two-dimensional mean-square displacement (MSD) of the PH domain from the two equilibrium simulations of the membrane-bound wild-type protein, which yielded values of *D* = 1.4 × 10^−8^ ± 0.3 × 10^−8^ cm^2^/s and 6.3 × 10^−8^ ± 2.2 × 10^−8^ cm^2^/s. These values compare reasonably well with the experimental value of *D* = 3.0 × 10^−8^ ± 0.3 × 10^−8^ cm^2^/s obtained by Knight and Falke, again suggesting that the simulations are able to reproduce experimentally observed behavior.

Kinetic binding parameters determined from previous experimental studies (Knight and Falke, 2009; Corbin et al., 2004) indicate that the value of *k*_off_ for wild-type GRP1-PH in mixed PC/PI(3,4,5)P_3_ lipid bilayers ranges from 0.38 to 1.60 s^-1^, depending upon experimental conditions and membrane composition. These data suggest that the mean lifetime of the complex, 1/*k*_off_, is of the order of seconds, and our MD simulations of the bound complex are therefore comparatively short. While we do not detect any distinctive changes in the global conformation of the protein over this period (Figure S3), this does not preclude the possibility that the degree of membrane insertion may change over longer timescales. In particular, we note that, in the crystal structure, the β3/β4 loop is twisted away from the binding cleft and, by extension, the membrane surface. As a result, relatively little membrane penetration is observed for this loop in the simulations. Although there is no evidence of this loop flexing and inserting more deeply into the bilayer during any of the simulations, this remains a possibility.

The pulling rates achievable in SMD (0.5 and 1.0 Å/ns in this case) are four orders of magnitude faster than experimental atomic force microscopy (AFM) rates (Contera et al., 2005). This fast pulling rate does induce some distortion in the protein structure (Figure S4), and the protein is capable of removing lipids from the bilayer at this velocity (Figure 4). However, subsequent removal of the force does lead to a partial recovery of the secondary structure. We also note that the approach used here is similar to that used to investigate of the force required to remove individual lipid molecules from a bilayer (Marrink et al., 1998) and that our pulling rate is comparable to or slower than that used in more recent studies of partitioning of anesthetics into lipid bilayers (Vemparala et al., 2006) and the unbinding of protein-ligand complexes (Zhang et al., 2006; Gräter et al., 2006). To assess the reliability of the SMD results, we ran SMD simulations at two different speeds (0.5 Å/ns and 1.0 Å/ns) for different lengths of time (80 and 40 ns) such that the distance moved by the protein remained the same. We still observe distortion in the protein secondary structure (Figure S4), and performing the same energy analysis as that shown in Figure 5 yields similar results (Figure S1).

By pulling the protein along the z axis perpendicular to the surface of the lipid bilayer, we have effectively defined the dissociation pathway of the protein-membrane complex a priori. This pathway of detachment of the protein from the surface of the membrane may not be the preferred dissociation mechanism in vivo, and it is likely that other dissociation pathways may exist. Here then, we have made use of SMD simply to study the notional response of the protein-membrane complex to an applied force. Accordingly, we restrict ourselves to making only qualitative observations about the relative strength and pattern of interactions between different regions of the protein and the lipid bilayer. We freely acknowledge that attempting to extract more quantitative information, such as free energies, would require much more extensive computations in order to reliably estimate these values. For a complex system such as this, it is expected that obtaining sufficiently well-converged potentials of mean force in order to draw meaningful conclusions would be extremely difficult, owing to the inevitable problems attaining adequate levels of sampling. SMD therefore provides a computationally straightforward way to elicit what is essentially a qualitative picture of how different regions of the protein may interact with the lipid bilayer.

A computational study of the behavior of PI(4,5)P_2_ in lipid bilayers published recently (Lupyan et al., 2010) found that the work required to extract a PI(4,5)P_2_ molecule from a 1,2-dipalmitoyl-*sn*-glycero-3-phosphocholine (DPPC) bilayer using SMD simulations was approximately three times greater than that required to remove a DPPC molecule from the same bilayer. The authors ascribed this behavior to the formation of a lipid microdomain around the PI(4,5)P_2_ molecule and rationalized this result by noting that the primary role of PI(4,5)P_2_ is thought to be the provision of a stable, membrane-bound anchor for signaling proteins at the plasma membrane. Our observation that the bound GRP1-PH-PI(3,4,5)P_3_ complex is able to extract POPC lipids when removed from the membrane surface during our SMD simulations, but that the PI(3,4,5)P_3_ molecule remains within the lipid bilayer, appears to be consistent with this result.

Several authors have reported that the presence of anionic lipids such as phosphatidylserine or other PI species increases the binding affinity of the bound complex (He et al., 2008; Corbin et al., 2004) and also that these anionic lipids exert a drag force, slowing the lateral diffusion of the complex at the membrane surface (Knight and Falke, 2009). Several candidate Arg and Lys residues close to the PI(3,4,5)P_3_ binding site may be responsible for sequestration of additional anionic lipids, though this possibility has not been explored here.

In the present work, we have shown that the three putative membrane-binding loops of GRP1-PH are all able to penetrate the lipid bilayer to a variable degree. Of these three loops, MD simulations of the wild-type protein predict that the β6/β7 loop penetrates to the greatest extent. This is supported by NMR data that showed a large change in chemical shift occurred in this region of the protein on binding to PI(3,4,5)P_3_-containing DPC micelles. Furthermore, during forcible dissociation of the complex by SMD simulations, the β6/β7 loop retained interactions with the bilayer for a longer period relative to the other two loops.

The NMR data presented in this paper provide further evidence that the binding of GRP1-PH is pH dependent, and support previous findings that suggested that H355 is crucial for successful binding and insertion. Protonation of this residue and the use of SMD simulations to pull the protein away from the bilayer revealed that the protonated H355 not only experienced a stronger interaction with PI(3,4,5)P_3_ but also contributed to a more pronounced interaction between the protein and the lipid bilayer. This apparent dual role for H355 tentatively suggests a possible explanation for the observed increase in the binding affinity on protonation of this residue.

Monolayer penetration experiments on GRP1-PH mutants showed that mutation of hydrophobic residues in the membrane binding loops led to a reduction in membrane insertion. This was in agreement with the results of MD simulations of the A346E mutant, which revealed that the protein experienced fewer nonpolar contacts with the lipid tails relative to the wild-type. Thus, while the initial diffusion encounter between the protein and the highly charged PI ligand is primarily governed by electrostatic interactions, taken together these results provide clear evidence that nonspecific interactions play a role in binding the GRP1 PH domain to the membrane surface and sustaining the bound complex. The work described above suggests a “dual-recognition” mechanism of interaction whereby the protrusion of the PI(3,4,5)P_3_ head group from the bilayer surface facilitates initial interactions between the PH domain and the target membrane, which are then reinforced through “deeper,” nonspecific contacts with the adjacent bilayer.

While the mutation of residues within the PI(3,4,5)P_3_ binding site has been shown to considerably reduce the binding affinity of GRP1-PH (Cronin et al., 2004; He et al., 2008), the observed reduction in membrane penetration in the mutants described above raises the possibility that mutations in other regions could also lead to a reduction in binding affinity and a corresponding loss of function. It is hoped that this more extensive description of the membrane-associated state of GRP1-PH, gleaned from the application of a variety of techniques to characterize the membrane-bound complex, may provide a route to link structural and biophysical studies through to system-level descriptions of signaling events at the plasma membrane (Bandara et al., 2009).

Recently, Knight et al. extended their studies of GRP1-PH to look at multimeric GRP1-PH domains (Knight et al., 2010). The authors again used TIRFM, this time to explore the collective diffusion at the membrane surface of multiple GRP1-PH domains tethered together. However, they also employed MD simulations as a complementary technique to aid in the analysis of their single-molecule experiments, which can otherwise be difficult to interpret. As with the work we have presented in this paper, the authors demonstrated that by using a combination of biophysical methods it was possible to probe complex biological processes in more detail than would be possible using a single technique. Knight et al. present some intriguing results, which suggest that diffusion of peripheral proteins at membrane surfaces is a more subtle phenomenon than was previously thought. While membrane diffusion has not been the primary focus of the current study, it is clear that further computational work in this area will be of considerable interest.

## Experimental Procedures

### Molecular Dynamics Simulations

MD simulations were performed using GROMACS version 3.3.3 (Lindahl et al., 2001) with the GROMOS96 43a1 forcefield (van Gunsteren et al., 1996). Bond lengths and angles were constrained using the LINCS algorithm (Hess et al., 1997) and a timestep of Δ*t* = 0.002 ps was used, writing atomic positions every 10 ps. The neighbor list was updated every ten steps. The 1.5 Å resolution structure of GRP1-PH complexed with I(1,3,4,5)P_4_ (PDB 1FGY (Lietzke et al., 2000)) was used in the simulations. Forcefield parameters for the I(1,3,4,5)P_4_ head group were generated as described in the Supplemental Experimental Procedures. The charge on the I(1,3,4,5)P_4_ head group was assumed to be −7, although in vivo the net charge of PI(3,4,5)P_3_ does, in fact, vary substantially (Kooijman et al., 2009). McLaughlin and co-workers point out that the net charge of PI(4,5)P_2_—and, by extension, PI(3,4,5)P_3_—is also likely to be influenced by the interaction with PI-binding proteins because protons bound to the head group may be displaced upon protein binding (McLaughlin et al., 2002). Despite this, when Lai et al. (2010) performed simulations of a membrane-bound C2 domain from protein kinase Cα (PKCα) in complex with PI(4,5)P_2_ they did not observe any change in the stability of the protein-membrane complex upon altering the net charge on PI(4,5)P_2_.

Simulations were performed under periodic boundary conditions and temperature was kept constant at 296 K by coupling the system to a heat bath using a Berendsen thermostat (Berendsen et al., 1984) with τ_T_ = 0.1 ps. Pressure was maintained at 1 atm using a Parrinello-Rahman barostat (Parrinello and Rahman, 1981; Nosé and Klein, 1983) and semi-isotropic pressure coupling with τ_p_ = 1 ps and a compressibility of 4.6 × 10^−5^ bar^-1^. Long-range electrostatics were treated using the particle mesh Ewald method (Darden et al., 1993) with a cutoff of 10 Å. To obtain the initial membrane-bound conformation of the protein, the I(1,3,4,5)P_4_ head group in complex with GRP1-PH from the crystal structure was overlaid onto a phosphatidylcholine head group of a lipid molecule close to the center of a preformed 1-palmitoyl,2-oleoyl-*sn*-glycero-3-phosphocholine (POPC) bilayer comprising 174 lipid molecules. The I(1,3,4,5)P_4_ head group was positioned such that the C1-C4 vector lay at an angle of approximately 40° to the membrane surface, while the C3-C5 vector lay at −17.5° to the membrane surface, within the ranges determined in a recent computational study of PI head group orientations in lipid bilayers (Li et al., 2009). The protein-ligand complex was then exchanged for the PC head group and solvent molecules were added using the GROMACS tools, employing the simple point charge (SPC) model for water (Hermans et al., 1984). After addition of the requisite number of ions to neutralize its net charge, the system was energy minimized for up to 1000 steps using the steepest descent algorithm and then sequentially equilibrated for a total of 3 ns. During the first 1 ns of equilibration, isotropic position restraints were applied to the protein and the ligand allowing the water and lipids to relax around the static complex. Restraints on the protein side chains were then removed, and equilibration continued for a further 1 ns, before continuing for a final 1 ns with all restraints removed bar those on the protein Cα atoms and the ligand atoms. Production runs were then performed for 100 ns to allow the protein to adopt its preferred orientation at the bilayer surface, and subsequent steered molecular dynamics (SMD) simulations were initiated from the conformation achieved after 50 ns of equilibrium MD simulation.

SMD simulations were carried out using the GROMACS pull code. A stiff, harmonic spring of force constant 500 pN/Å was attached to the center of mass of the protein, though the protein remained free to rotate about its center of mass. Another, identical spring was attached to the center of mass of the lipid molecules in the bilayer to maintain the integrity and planarity of the bilayer as the protein was pulled away. The spring attached to the protein was then retracted parallel to the z axis at a rate of 1.0 Å/ns over a period of 40 ns or 0.5 Å/ns over a period of 80 ns.

Lateral diffusion constants were estimated by first removing the center-of-mass motion of the combined protein and lipid bilayer and then calculating the MSD of the protein in the *xy* plane, such that the motion of the protein was measured relative to the center-of-mass of the membrane. The value of *D* was extracted by fitting to the MSD over the first 50 ns of the simulation trajectory where it is approximately linear using the GROMACS tools, and subsequently using the Einstein relation in two dimensions (Hansen and McDonald, 2006). Mutations were performed using VMD (Humphrey et al., 1996) and molecular graphics were generated using PyMOL (DeLano, 2002).

### Expression and Purification of GRP1 PH Domain

The ^15^N-labeled PH domain of human GRP1 (residues 261–385; pRSET A vector) was expressed in *Escherichia coli* Rosetta in minimal media supplemented with ^15^NH_4_Cl (Isotec). Bacteria were harvested by centrifugation after induction with IPTG (0.1 mM) and lysed by sonication. The 6xHis fusion protein was purified on a Talon-resin column (Clontech Laboratories, Inc.). The His tag was cleaved with EKMax (Invitrogen), and the GRP1 PH domain was further purified by ion exchange chromatography on HiTrap SP HP column (Amersham) in Bis-Tris ion exchange buffer (pH 6.5) and concentrated in Millipore concentrators as described in He et al. (2008).

### NMR Spectroscopy

NMR spectra were recorded at 25°C on a Varian INOVA 600 MHz spectrometer. The ^1^H, ^15^N heteronuclear single quantum coherence (HSQC) spectra of 0.2 mM ^15^N-labeled GRP1 PH domain were collected using 1024 t1 increments of 2048 data points, 128 number of increments, and spectral widths of 8000 and 2000 Hz in the ^1^H and ^15^N dimensions, respectively. Association with DPC micelles at pH 6.0, 6.8, and 7.4 was characterized by monitoring chemical shift changes in the ^1^H, ^15^N HSQC spectra of the C_4_-PI(3,4,5)P_3_ (Echelon Biosciences, Inc.)-bound PH domain at a 2:1 lipid-to-protein ratio as DPC was gradually added up to 286 mM (or 5.1 mM micellar). Micellar concentration corresponds to the solution concentration of intact micelles and is obtained by dividing the value of the detergent molecular concentration by 56, an average aggregation number of DPC (Lauterwein et al., 1979).

### PCR Mutagenesis of the GRP1 PH Domain

Site-directed mutagenesis of the GRP1 PH domain was performed using a QuikChange kit (Stratagene). The sequences of the V278E, Y298E, A346E, and V351E constructs were confirmed by DNA sequencing.

### Monolayer Measurements

The penetration of the wild-type and mutant GRP1 PH domains into the phospholipid monolayer was investigated by measuring the change in surface pressure π of invariable surface area during addition of the proteins. The experiments were performed using a 1 ml circular Teflon trough and wire probe connected to a Kibron MicroTrough X (Kibron, Inc., Helsinki). A lipid monolayer containing various combinations of phospholipids was spread onto the subphase composed of 10 mM HEPES/0.16 M KCl (pH 7.4), until the desired initial surface pressure π_0_ was reached. After stabilization of the signal (∼5 min), 10 μg of protein was injected into the subphase through a hole in the wall of the trough. The surface pressure change Δπ was monitored for 45 min. The Δπ value reached a maximum after 30 min in all experiments.

## Supplemental Information

Document S1. Four Figures and Supplemental Experimental Procedures

## References

[bib1] Arkhipov A., Yin Y., Schulten K. (2008). Four-scale description of membrane sculpting by BAR domains. Biophys. J..

[bib2] Bandara S., Schlöder J.P., Eils R., Bock H.G., Meyer T. (2009). Optimal experimental design for parameter estimation of a cell signaling model. PLoS Comput. Biol..

[bib3] Berendsen H.J.C., Postma J.P.M., van Gunsteren W.F., DiNola A., Haak J.R. (1984). Molecular dynamics with coupling to an external bath. J. Chem. Phys..

[bib4] Blood P.D., Voth G.A. (2006). Direct observation of Bin/amphiphysin/Rvs (BAR) domain-induced membrane curvature by means of molecular dynamics simulations. Proc. Natl. Acad. Sci. USA.

[bib5] Cantley L.C. (2002). The phosphoinositide 3-kinase pathway. Science.

[bib6] Cho W., Stahelin R.V. (2005). Membrane-protein interactions in cell signaling and membrane trafficking. Annu. Rev. Biophys. Biomol. Struct..

[bib7] Contera S.A., Lemaître V., de Planque M.R.R., Watts A., Ryan J.F. (2005). Unfolding and extraction of a transmembrane α-helical peptide: dynamic force spectroscopy and molecular dynamics simulations. Biophys. J..

[bib8] Corbin J.A., Dirkx R., Falke J.J. (2004). GRP1 pleckstrin homology domain: activation parameters and novel search mechanism for rare target lipid. Biochemistry.

[bib9] Cronin T.C., DiNitto J.P., Czech M.P., Lambright D.G. (2004). Structural determinants of phosphoinositide selectivity in splice variants of Grp1 family PH domains. EMBO J..

[bib10] Darden T., York D., Pedersen L. (1993). Particle mesh Ewald: an N log N method for Ewald sums in large systems. J. Chem. Phys..

[bib11] DeLano, W.L. (2002). The PyMOL Molecular Graphics System, Version 1.3, Schrödinger, LLC.

[bib12] DiNitto J.P., Delprato A., Lee M.-T., Cronin T., Huang S., Guilherme A., Czech M.P., Lambright D.G. (2007). Structural basis and mechanism of autoregulation in 3-phosphoinositide-dependent Grp1 family Arf GTPase exchange factors. Mol. Cell.

[bib13] D'Souza-Schorey C., Chavrier P. (2006). ARF proteins: roles in membrane traffic and beyond. Nat. Rev. Mol. Cell Biol..

[bib14] Ferguson K.M., Kavran J.M., Sankaran V.G., Fournier E., Isakoff S.J., Skolnik E.Y., Lemmon M.A. (2000). Structural basis for discrimination of 3-phosphoinositides by pleckstrin homology domains. Mol. Cell.

[bib15] Flesch F.M., Yu J.W., Lemmon M.A., Burger K.N.J. (2005). Membrane activity of the phospholipase C-δ 1 pleckstrin homology (PH) domain. Biochem. J..

[bib16] Gräter F., de Groot B.L., Jiang H., Grubmüller H. (2006). Ligand-release pathways in the pheromone-binding protein of *Bombyx mori*. Structure.

[bib17] Grzesiek S., Stahl S.J., Wingfield P.T., Bax A. (1996). The CD4 determinant for downregulation by HIV-1 Nef directly binds to Nef. Mapping of the Nef binding surface by NMR. Biochemistry.

[bib18] Hansen J.-P., McDonald I.R. (2006). Theory of Simple Liquids.

[bib19] Haslam R.J., Koide H.B., Hemmings B.A. (1993). Pleckstrin domain homology. Nature.

[bib20] He J., Haney R.M., Vora M., Verkhusha V.V., Stahelin R.V., Kutateladze T.G. (2008). Molecular mechanism of membrane targeting by the Grp1 PH domain. J. Lipid Res..

[bib21] Hermans J., Berendsen H.J.C., van Gunsteren W.F., Postma J.P.M. (1984). A consistent empirical potential for water-protein interactions. Biopolymers.

[bib22] Hess B., Bekker H., Berendsen H.J.C., Fraaije J.G.E.M. (1997). LINCS: a linear constraint solver for molecular simulations. J. Comput. Chem..

[bib23] Humphrey W., Dalke A., Schulten K. (1996). VMD - Visual Molecular Dynamics. J. Mol. Graph..

[bib24] Jaud S., Tobias D.J., Falke J.J., White S.H. (2007). Self-induced docking site of a deeply embedded peripheral membrane protein. Biophys. J..

[bib25] Kalli A.C., Wegener K.L., Goult B.T., Anthis N.J., Campbell I.D., Sansom M.S.P. (2010). The structure of the talin/integrin complex at a lipid bilayer: An NMR and MD simulation study. Structure.

[bib26] Kavran J.M., Klein D.E., Lee A., Falasca M., Isakoff S.J., Skolnik E.Y., Lemmon M.A. (1998). Specificity and promiscuity in phosphoinositide binding by pleckstrin homology domains. J. Biol. Chem..

[bib27] Klarlund J.K., Guilherme A., Holik J.J., Virbasius J.V., Chawla A., Czech M.P. (1997). Signaling of phosphoinositide-3,4,5-trisphosphate through proteins containing pleckstrin and Sec7 homology domains. Science.

[bib28] Klarlund J.K., Tsiaras T., Holik J.J., Chawla A., Czech M.P. (2000). Distinct polyphosphoinositide binding selectivities for pleckstrin homology domains of GRP1 like proteins based on diglycine versus triglycine motifs. J. Biol. Chem..

[bib29] Knight J.D., Falke J.J. (2009). Single molecule fluorescence studies of a PH domain: New insights into the membrane docking reaction. Biophys. J..

[bib30] Knight J.D., Lerner M.G., Marcano-Velázquez J.G., Pastor R.W., Falke J.J. (2010). Single molecule diffusion of membrane-bound proteins: Window into lipid contacts and bilayer dynamics. Biophys. J..

[bib31] Kooijman E.E., King K.E., Gangoda M., Gericke A. (2009). Ionization properties of phosphatidylinositol polyphosphates in mixed model membranes. Biochemistry.

[bib32] Lai C.-L., Landgraf K.E., Voth G.A., Falke J.J. (2010). Membrane docking geometry and target lipid stoichiometry of membrane-bound PKCα C2 domain: A combined molecular dynamics and experimental study. J. Mol. Biol..

[bib33] Lauterwein J., Bösch C., Brown L.R., Wüthrich K. (1979). Physicochemical studies of the protein-lipid interactions in melittin-containing micelles. Biochim. Biophys. Acta.

[bib34] Lemmon M.A. (2008). Membrane recognition by phospholipid-binding domains. Nat. Rev. Mol. Cell Biol..

[bib35] Lemmon M.A., Ferguson K.M., Schlessinger J. (1996). PH domains: diverse sequences with a common fold recruit signaling molecules to the cell surface. Cell.

[bib36] Li Z., Venable R.M., Rogers L.A., Murray D., Pastor R.W. (2009). Molecular dynamics simulations of PIP2 and PIP3 in lipid bilayers: determination of ring orientation, and the effects of surface roughness on a Poisson-Boltzmann description. Biophys. J..

[bib37] Lietzke S.E., Bose S., Cronin T., Klarlund J.K., Chawla A., Czech M.P., Lambright D.G. (2000). Structural basis of 3-phosphoinositide recognition by pleckstrin homology domains. Mol. Cell.

[bib38] Lindahl E., Sansom M.S.P. (2008). Membrane proteins: molecular dynamics simulations. Curr. Opin. Struct. Biol..

[bib39] Lindahl E., Hess B., van der Spoel D. (2001). GROMACS 3.0: a package for molecular simulation and trajectory analysis. J. Mol. Model..

[bib40] Lupyan D., Mezel M., Logothetis D.E., Osman R. (2010). A molecular dynamics investigation of lipid bilayer perturbation by PIP2. Biophys. J..

[bib41] Manna D., Albanese A., Park W.S., Cho W. (2007). Mechanistic basis of differential cellular responses of phosphatidylinositol 3,4-bisphosphate- and phosphatidylinositol 3,4,5-trisphosphate-binding pleckstrin homology domains. J. Biol. Chem..

[bib42] Manna D., Bhardwaj N., Vora M.S., Stahelin R.V., Lu H., Cho W. (2008). Differential roles of phosphatidylserine, PtdIns(4,5)P_2_ and PtdIns(3,4,5)P_3_ in plasma membrane targeting of C2 domains. J. Biol. Chem..

[bib43] Marrink S.-J., Berger O., Tieleman D.P., Jähnig F. (1998). Adhesion forces of lipids in a phospholipid membrane studied by molecular dynamics simulations. Biophys. J..

[bib44] Mayer B.J., Ren R., Clark K.L., Baltimore D. (1993). A putative modular domain present in diverse signaling proteins. Cell.

[bib45] McLaughlin S., Wang J., Gambhir A., Murray D. (2002). PIP2 and proteins: Interactions, organization, and information flow. Annu. Rev. Biophys. Biomol. Struct..

[bib46] Nosé S., Klein M.L. (1983). Constant pressure molecular dynamics for molecular systems. Mol. Phy..

[bib47] Ohkubo Y.Z., Tajkhorshid E. (2008). Distinct structural and adhesive roles of Ca^2+^ in membrane binding of blood coagulation factors. Structure.

[bib48] Parrinello M., Rahman A. (1981). Polymorphic transitions in single crystals: A new molecular dynamics method. J. Appl. Phy..

[bib49] Psachoulia E., Sansom M.S.P. (2008). Interactions of the pleckstrin homology domain with phosphatidylinositol phosphate and membranes: Characterization via molecular dynamics simulations. Biochemistry.

[bib50] Psachoulia E., Sansom M.S.P. (2009). PX- and FYVE-mediated interactions with membranes: Simulation studies. Biochemistry.

[bib51] Stahelin R.V. (2009). Lipid binding domains: More than simple lipid effectors. J. Lipid Res..

[bib52] Tague S.E., Muralidharan V., D'Souza-Schorey C. (2004). ADP-ribosylation factor 6 regulates tumor cell invasion through the activation of the MEK/ERK signaling pathway. Proc. Natl. Acad. Sci. USA.

[bib53] van Gunsteren, W.F., Billeter, S.R., Eising, A.A., Hünenberger, P.H., Krüger, P., Mark, A.E., Scott, W., and Tironi, I.G. (1996). Biomolecular Simulation: The GROMOS96 Manual and User Guide. Zurich: Biomos & Hochschulverlag AG an der ETH Zurich.

[bib54] Vemparala S., Saiz L., Eckenhoff R.G., Klein M.L. (2006). Partitioning of anaesthetics into a lipid bilayer and their interaction with membrane-bound peptide bundles. Biophys. J..

[bib55] Wymann M.P., Schneiter R. (2008). Lipid signaling in disease. Nat. Rev. Mol. Cell Biol..

[bib56] Zhang D., Gullingsrud J., McCammon J.A. (2006). Potentials of mean force for acetylcholine unbinding from the alpha7 nicotinic acetylcholine receptor ligand-binding domain. J. Am. Chem. Soc..

