# Supplementary material for: Biophysical and Computational Studies of Membrane Penetration by the GRP1 Pleckstrin Homology Domain
Source: Structure. 2011 Sep 7;19(9-16):1338–46. doi: 10.1016/j.str.2011.04.010 (PMC3173609; doi:10.1016/j.str.2011.04.010)
Supplement: Document S1. Four Figures and Supplemental Experimental Procedures [file mmc1.pdf]

Structure *19*

Supplemental Information

Biophysical and Computational Studies of Membrane Penetration by the GRP1 Pleckstrin Homology Domain

Craig N. Lumb, Ju He, Yi Xue, Phillip J. Stansfeld, Robert V. Stahelin, Tatiana G. Kutateladze, and Mark S.P. Sansom

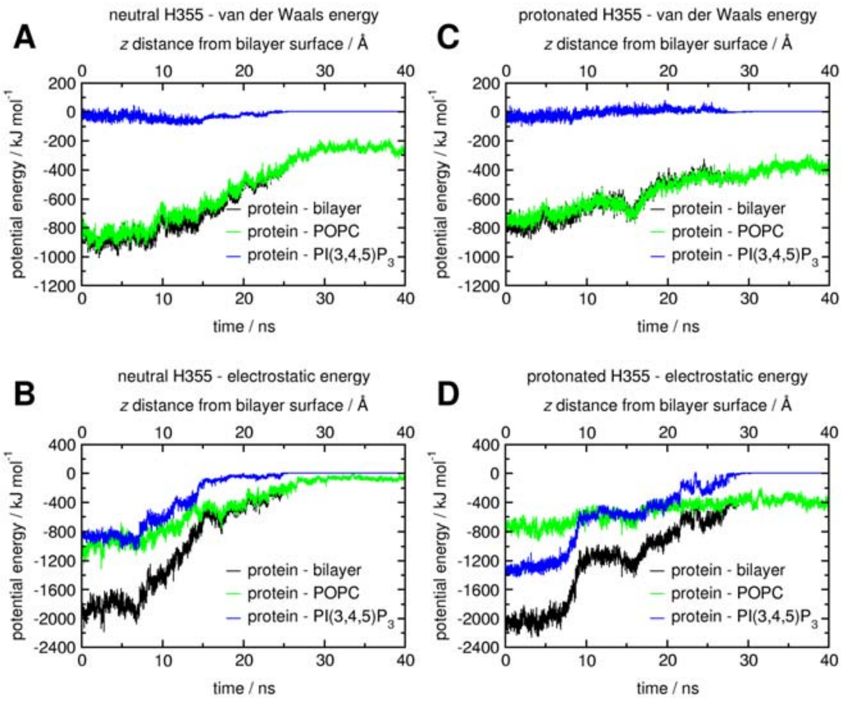

Figure S1, related to Figure 5

Short-range components of the van der Waals and electrostatic contributions to the potential energy of the protein-membrane complex calculated from the shorter SMD simulations, duration 40 ns and a pulling rate of 1.0 Å/ns. The van der Waals potential energy is shown for (A) the system with neutral H355 and (B) the system with H355 in a protonated state. Electrostatic potential energies are shown in (C) for neutral H355 and in (D) for H355 in a protonated state. The traces are qualitatively similar in shape to those calculated for the slower SMD simulations, 80 ns duration and a pulling rate of 0.5 Å/ns.

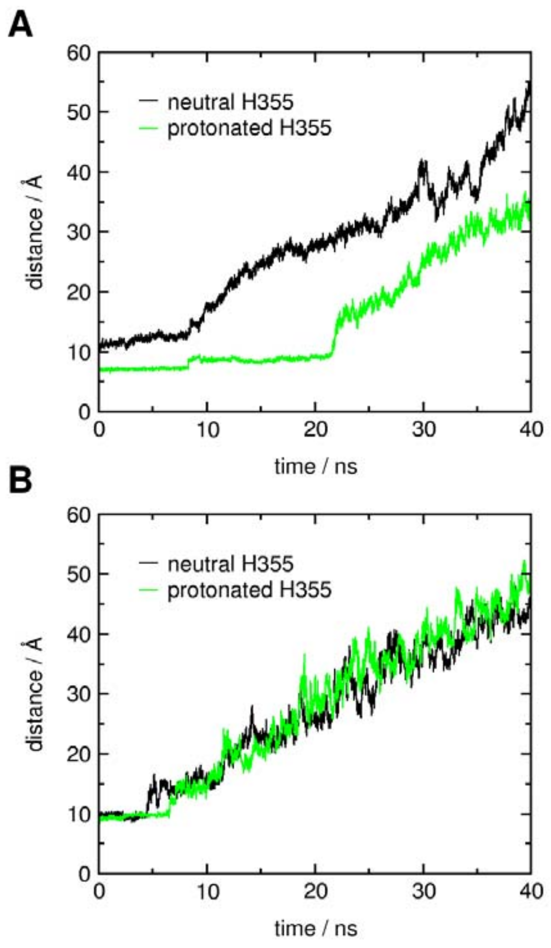

Figure S2, related to Figure 5

Interactions of the protein with both PI(3,4,5)P_3 and the lipids in the membrane are enhanced by protonation of H355. (A) Distance between the center of mass of the I(1,3,4,5)P_4 headgroup and the center of mass of H355 in the membrane-bound SMD simulations for both the neutral H355 case (black) and the protonated H355 case (green). The protonated H355 residue appears to act as a 'latch', prolonging the interaction between the protein and the I(1,3,4,5)P_4 headgroup as the protein is pulled away. (B) The same measure is shown, this time for the SMD simulations carried out with the protein and isolated I(1,3,4,5)P_4 headgroup in solution, with no bilayer present. The 'latching' behavior seen in the membrane-bound simulations is still observed, but is a much weaker effect in the absence of the lipid bilayer, suggesting that protonation also favours a more substantial interaction with the membrane.

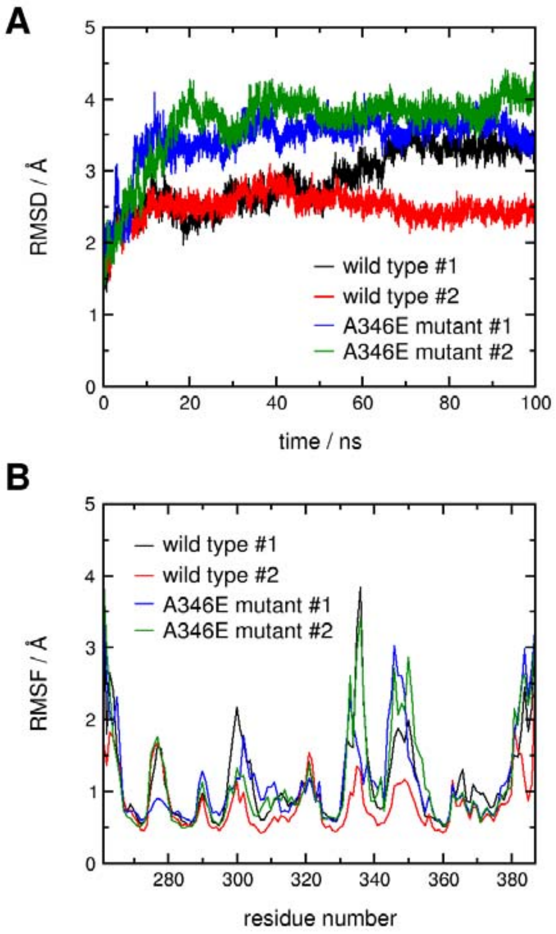

Figure S3, related to Figure 2

Conformational drift of bound GRP1-PH during the equilibrium MD simulations. (A) Root mean square deviation and (B) root mean square fluctuation of the protein Cα atoms over the 100 ns trajectories. The wild type simulations are shown in black and red, while the simulations of the A346E mutant are shown in blue and green.

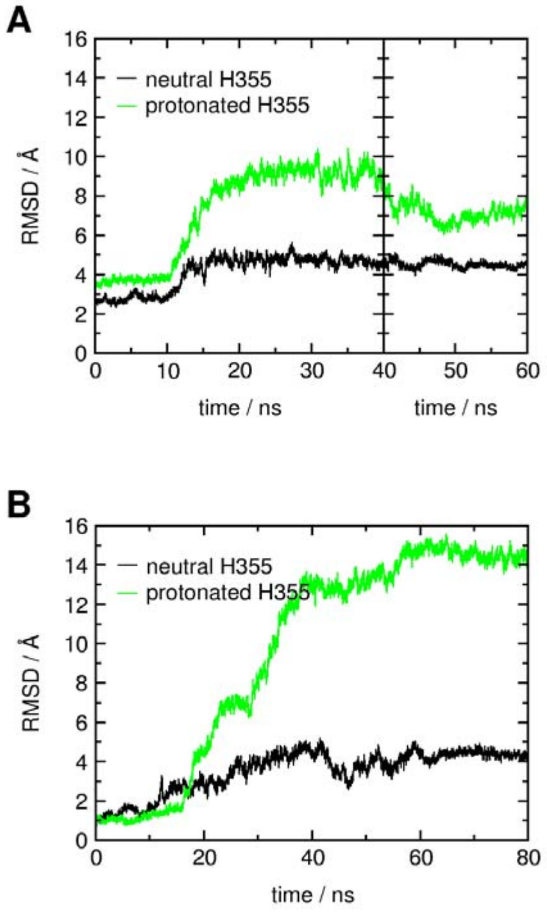

Figure S4, related to Figure 4

RMSD of the protein Cα atoms over the course of the SMD trajectories (A) RMSD of the protein Cα atoms for the faster SMD trajectory, duration 40 ns and a pulling rate of 1.0 Å/ns, and the resulting effect on the RMSD when the force was removed and the system was simulated under equilibrium conditions for a further 20 ns. There is considerable distortion induced in the secondary structure during the SMD simulations, particularly in the case where H355 is protonated, reflecting the higher affinity binding to the membrane. Removing the applied force and continuing the simulation leads to partial recovery of the secondary structure. (B) RMSD of the protein Cα atoms for the slower SMD trajectory, duration 80 ns and a pulling rate of 0.5 Å/ns. Distortion is still induced in the protein structure even at this slower pulling rate.

SUPPLEMENTAL EXPERIMENTAL PROCEDURES

Molecular dynamics simulations

Forcefield parameters for the I(1,3,4,5)P_4 headgroup were obtained using PRODRG (Schuettelkopf and van Aalten, 2004), and atomic partial charges were refined by performing geometry optimization and a subsequent single-point energy calculation on the headgroup using the GAUSSIAN package (Frisch et al., 2004), with the 6-31G** split valence basis set and the B3LYP hybrid functional (Becke, 1993; Lee et al., 1988; Stephens et al., 1994) in a similar vein to Blood *et al.* (Blood et al., 2008) and Lupyan *et al.,* (Lupyan et al., 2010).

SUPPLEMENTAL REFERENCES

Becke, A.D. (1993). Density-functional thermochemistry. III. The role of exact exchange. J. Chem. Phys. *98*, 5648-5652.

Blood, P.D., Swenson, R.D., and Voth, G.A. (2008). Factors influencing local membrane curvature induction by N-BAR domains as revealed by molecular dynamics simulations. Biophys. J. *95*, 1866-1876.

Frisch, M.J., Trucks, G.W., Schlegel, H.B., Scuseria, G.E., Robb, M.A., Cheeseman, J.R., Montgomery Jr, J.A., Vreven, T., Kudin, K.N., Burant, J.C., Millam, J.M., Iyengar, S.S., Tomasi, J., Barone, V., Mennucci, B., Cossi, M., Scalmani, G., Rega, N., Petersson, G.A., Nakatsuji, H., Hada, M., Ehara, M., Toyota, K., Fukuda, R., Hasegawa, J., Ishida, M., Nakajima, T., Honda, Y., Kitao, O., Nakai, H., Klene, M., Li, X., Knox, J.E., Hratchian, H.P., Cross, J.B., Bakken, V., Adamo, C., Jaramillo, J., Gomperts, R., Stratmann, R.E., Yazyev, O., Austin, A.J., Cammi, R., Pomelli, C., Ochterski, J.W., Ayala, P.Y., Morokuma, K., Voth, G.A., Salvador, P., Dannenberg, J.J., Zakrzewski, V.G., Dapprich, S., Daniels, A.D., Strain, M.C., Farkas, O., Malick, D.K., Rabuck, A.D.,

Raghavachari, K., Foresman, J.B., Ortiz, J.V., Cui, Q., Baboul, A.G., Clifford, S., Cioslowski, J., Stefanov, B.B., Liu, G., Liashenko, A., Piskorz, P., Komaromi, I., Martin, R.L., Fox, D.J., Keith, T., Al-Laham, M.A., Peng, C.Y., Nanayakkara, A., Challacombe, M., Gill, P.M.W., Johnson, B., Chen, W., Wong, M.W., Gonzalez, C. and Pople, J.A. (2004) Gaussian 03, Revision E.01. Gaussian, Inc., Wallingford CT

Lee, C., Yang, W., and Parr, R.G. (1988). Development of the Colle-Salvetti correlation-energy formula into a functional of the electron density. Phys. Rev. B *37*, 785-789.

Lupyan, D., Mezel, M., Logothetis, D.E., and Osman, R. (2010). A molecular dynamics investigation of lipid bilayer perturbation by PIP2. Biophys. J. *98*, 240-247.

Schuettelkopf, A.W., and van Aalten, D.M.F. (2004). PRODRG - a tool for high-throughput crystallography of protein-ligand complexes. Acta. Crystallogr. *D60*, 1355-1363.

Stephens, P.J., Devlin, F.J., Chabalowski, C.F., and Frisch, M.J. (1994). *Ab initio* calculation of vibrational absorption and circular dichroism spectra using density functional force fields. J. Phys. Chem. *98*, 11623-11627.
